# Clinical Analysis of Childhood Acute Lymphoblastic Leukemia With Epilepsy Seizures

**DOI:** 10.3389/fneur.2022.824268

**Published:** 2022-05-10

**Authors:** Rui Li, Ji-Hong Tang, Bing-Bing Zhang, Xiao-Yan Shi, Yuan-Yuan Dai, Rui Qu

**Affiliations:** ^1^Department of Neurology, Children's Hospital of Soochow University, Suzhou, China; ^2^Department of Pediatric, Affiliated Hospital of Xuzhou Medical University, Xuzhou, China

**Keywords:** ALL, epilepsy, posterior reversible encephalopathy syndrome, abnormal white brain matter, prognosis

## Abstract

**Objective:**

In order to analyze the clinical characteristics of epileptic seizures in children with acute lymphoblastic leukemia (ALL) during treatment.

**Methods:**

The clinical and imaging data of children diagnosed as ALL with epilepsy seizures from January 2013 to December 2020 were retrospectively analyzed.

**Results:**

A total of 2217 children with ALL were admitted during the study, of whom 229 (10.33%) had epileptic seizures after ALL treatment. Among them, 45 (19.65%) were in the high-risk group and 184 (80.35%) were in the low-risk group. Epileptic seizures mainly occurred in the induction remission period (24.02%), maintenance treatment period (25.33%) and after bone marrow transplantation (21.40%). The common causes were MTX-related demyelinating encephalopathy (34.06%) and reversible posterior encephalopathy syndrome (PRES) (25.3%). The first symptom was mainly convulsion (34.50%). The first attack had a comprehensive attack and partial attack. Most patients stop themselves. 30 cases (13.10%) had acute recurrence of epilepsy (recurrence within 3 months after the first attack), and 49 cases (25.76%) had neurological dysfunction after follow-up. 36 cases developed symptomatic epilepsy. Among the 130 children who completed the follow-up, 78 (60.00%) had no obvious neurological sequelae, and 52 (40.0%) had neurological sequelae. Among the 52 cases, there were 34 cases of mild sequelae and 18 cases of severe sequelae, including 8 cases of epilepsy combined with cognitive impairment.

**Conclusion:**

Epileptic seizure is a common neurological complication during ALL treatment. The etiology and associated manifestations of the first epileptic seizure are diverse. Early neuroimaging and EEG examination are helpful for early diagnosis and treatment.

## Introduction

The incidence of acute leukemia in children is on the rise in China, with an annual incidence of 4/100 000. More than 50 000 new childhood leukemias are added each year, ranking first in children with malignant tumors and becoming the number one killer threatening children's health (1). Acute lymphoblastic leukemia (ALL) accounted for 75% of all leukemias and 25% of all malignant tumors in children (2). In the past 20 years, the treatment of childhood with ALL has achieved remarkable results, and the treatment plan is also very mature. The disease-free survival rate of children with ALL in developed countries is close to 80%-85%, and the 5-year long-term survival rate of all children in China is more than 85%. ALL has become a curable malignant disease (3). With the continuous improvement of ALL treatment, the survival rate of ALL children was significantly improved. However, complications of central nervous system during acute gonorrhea therapy remain a challenging clinical problem. With the strengthening of chemotherapy, the prognosis of ALL was significantly improved, and the incidence of adverse events and the degree of physical injury after chemotherapy were also significantly increased. Therapeutic regimens containing intrathecal methotrexate (MTX) injections, for example, often lead to neurotoxicity (4, 5). In particular, epileptic seizures are more common. According to statistics, epileptic seizures occurred in 8 ~ 13% of ALL patients during treatment (6). There is a higher risk of late neurological complications in ALL patients with epilepsy than those without epilepsy, leading to symptomatic epilepsy and mental retardation (7). It is not only beneficial to timely and effective treatment and avoid irreversible damage, but also affects the choice of follow-up chemotherapy and the prognosis of nervous system in children with ALL. In this study, 236 cases of ALL children with epileptic seizures admitted to Suzhou Children' s Hospital from 2011 to 2020 were collected. The clinical manifestations, imaging, EEG and other data of epileptic seizures in children with acute lymphoblastic leukemia were analyzed to improve the clinical understanding of childhood ALL with epileptic seizures.

## Patients and Methods

### Population

The clinical data of children with ALL and epileptic seizures admitted and diagnosed in the Department of Blood Tumor, Children' s Hospital Affiliated to Suzhou University from January 2013 to June 2020 were retrospectively analyzed.

Inclusion criteria: ①Age 7 months ~ 14 years; ②ALL initially diagnosed; ③Treated in accordance with the Chinese Childhood Leukemia Cooperation Group ALL2008 Programme (CCLG-ALL2008 Programme) (8) or the Chinese Anti-Cancer Association Pediatric Oncology Committee ALL 2015 Programme (CCCG-ALL 2015 Programme) (9); ④Epileptic seizures occurred for the first time after chemotherapy.

Exclusion criteria: ①Epilepsy or epilepsy syndrome has been diagnosed before ALL; ②previous congenital diseases or genetic metabolic diseases; ③Patients with epilepsy as the first symptom, and then diagnosed as ALL.

This study was approved by the Hospital Medical Ethics Committee (No. 2021 CS 142). The guardian of the child was informed and signed the consent.

### Research Method

#### Clinical Data Collection and Follow-Up

Clinical data were collected during hospitalization and follow-up, including gender, age, seizure time and clinical manifestations, imaging examination, EEG results. All patients were followed up, 130 cases completed the follow-up. Fifty nine cases were followed up by neurology, 57 cases were followed up by telephone, and follow-up of 14 patients with recurrent seizures Visit. The shortest discharge time of 130 patients was 6 months and the longest was 7 years. The termination time of follow-up was December 31, 2020.

#### Diagnostic Criteria for Symptomatic Epilepsy

Epilepsy seizure is different from epilepsy. Symptomatic epilepsy refers to the occurrence of epileptic seizures during the course of disease, which is consistent with the definition of clinical practicability of epilepsy. Clinical practicability definition of epilepsy (10, 11): at least two non-inducible seizures (seizures with no clear acute inducement), and the interval between two seizures > 24 h. The risk of re-epileptic seizures in the next 10 years is comparable to the risk of recurrence after two non-epileptic seizures (at least 60%), and any of the following risk factors can be considered for recurrence. EEG showed typical epileptic discharge, and the discharge was correlated with epileptic seizures; 2 Neuroimaging showed abnormal brain structure, which was correlated with epileptic seizures; Significant brain dysfunction, such as mental retardation, focal signs (limb paralysis, limb sensory disturbance, aphasia, blindness), or long-term symptomatic causes (static brain lesions such as previous brain injury or congenital cortical dysplasia).

#### Evaluation of Sequelae in ALL Children With Epilepsy Seizures

The global burden of disease-disease control priorities project (GBD-DCPP) scale was used to evaluate the sequelae of 30 children who completed the follow-up (12).

### Statistical Analysis

SPSS23 software was used for statistical analysis. The measurement data were not subject to normal distribution and represented by median. The enumeration data are represented by n and constituent ratio.

## Results

### Characteristics of Primary Seizures

January 2013 to June 2020, a total of 2217 children were diagnosed with ALL. There were 229 ALL children with epilepsy seizures. The incidence was 10.33%. There are 146 males and 93 females. The age of onset ranged from 5 months to 16 years with a median age of 9 years. Among them, three cases were under 1 year old (1.31%), 174 cases were 1~10 years old (75.98%), 52 cases were 10 years old (22.71%). The proportion of epileptic seizures in ALL children during induction remission, maintenance treatment and after bone marrow transplantation were high. The constituent ratios were 24.02, 25.33, and 21.40%. Seventy eight cases (34.06%) were diagnosed as MTX related demyelinating encephalopathy. Twenty nine cases (12.66%) were diagnosed as central nervous system leukemia (CNSL). 58 cases (25.33%) were diagnosed as reversible posterior encephalopathy syndrome (PRES). The primary symptoms were mainly convulsion (34.50%). In 229 ALL children with epilepsy seizures, 127 cases had comprehensive seizures (55.46%) and 102 cases had local seizures (44.54%). Of the 127 cases with comprehensive seizures, 36 cases were persistent (28.35%). Thirty cases (13.10%) had acute recurrence of epilepsy (recurrence within 3 months after the first attack). Fourty nine cases (25.76%) had neurological abnormalities after follow-up. 36 cases developed symptomatic epilepsy (Table 1).

**Table 1:** Clinical characteristics of primary epilepsy seizure in ALL children.

**Variable**	**Classification**	***n***	**Constituent ratio**
Sex	Male	146	64.68%
	Female	83	35.32%
Age of first CNS symptom (years)	<1	3	1.31%
	1~10	174	75.98%
	>10	52	22.71%
Treatment	2015	108	48.51%
	2008	121	51.49%
Primary epilepsy seizure period	Induced remission stage	55	24.02%
	Early intensive treatment period	20	8.73%
	consolidation period	10	4.37%
	Delayed intensive treatment period	17	7.42%
	Maintenance treatment period	58	25.33%
	Mitigation period after chemotherapy	6	2.62%
	Retreatment period of recurrence	6	2.62%
	Before bone marrow transplantation	8	3.49%
	After bone marrow transplantation	49	21.40%
Diagnosis	MTX related demyelinating encephalopathy	78	34.06%
	CNSL	29	12.66%
	Reversible posterior brain syndrome (PRES)	58	25.33%
	Cerebral hernia	9	3.93%
	Cerebral hemorrhage	11	4.80%
	Unknown	44	19.22%
First symptom	Convulsions	79	34.50%
	Blurred vision	31	13.54%
	Fever	21	9.17%
	Vomit	24	10.48%
	Headache	23	10.04%
	Other symptoms	51	22.27%
Seizure degree	Comprehensive seizures	127	
	Continuous state	36	28.35%
	Stop by itself	81	71.65%
	Focal seizures	102	
	Continuous state	27	26.47%
	Stop by itself	75	73.53
Prognosis	Clinical recovery	170	74.24%
	Acute recurrence of epilepsy	30	13.10%
	Neurological sequelae	49	21.68%
	Symptomatic epilepsy	36	15.72%

### Factors Influencing Initial Seizures

There were 229 ALL children with epilepsy seizures. There were 45 (19.65%) cases in the high-risk group and 184 (80.35%) cases in the low-risk group of leukemia. 11 (4.80%) patients found CNSL when diagnosed with ALL. 28 (12.23%) cases had organic damage of central nervous system before the first attack (Table 2).

**Table 2:** Factors of primary epilepsy seizures.

**Factors**	**Classification**	***n***	**Constituent ratio**
Risk of leukemia	High-risk	45	19.65%
	Medium-low-risk	184	80.35%
CNSL	Existence at diagnosis	11	4.80%
	Existence at attack	18	7.86%
	No	200	87.34%
Organic damage of central nervous system before seizures	Yes	28	12.23%
	No	201	87.77%

### Etiology Diagnosis of Primary Epilepsy Seizures

Thirty one cases (56.36%) of ALL patients with induced remission seizures were related to PRES. the main cause of epilepsy during early intensive treatment was MTX—related demyelination (75.00%). Epileptic seizures in 38 ALL patients (65.52%) during maintenance treatment were also mainly associated with MTX—related demyelination. Epilepsy caused by CNSL occurs mainly during after bone marrow transplantation (Table 3).

**Table 3:** Etiology diagnosis of primary epilepsy seizures.

**Period**	***n***	**MTX**	**CNSL**	**PRES**	**Cerebral hernia**	**Cerebral hemorrhage**
Induced remission stage	55	10	7	31	2	4
Early intensive treatment period	20	15	4	1	0	0
Consolidation period	10	1	0	3	0	0
Delayed intensive treatment period	17	12	0	0	0	0
Maintenance treatment period	58	38	0	10	0	6
Mitigation period after chemotherapy	6	0	0	3	1	0
Retreatment period of recurrence	6	0	0	0	0	0
Before bone marrow transplantation	8	0	2	0	0	0
After bone marrow transplantation	49	2	16	10	6	0

### Follow-Up and Prognosis

Among the 130 children who completed the follow-up, 36 cases (26.7%) developed symptomatic epilepsy and were treated with antiepileptic drugs. Among them, 21 cases were treated with Levetiracetam alone and five cases were treated with Levetiracetam combined with Topiramate, and the seizures were controlled. six cases still recurred after treatment with Levetiracetam alone, and were treated with topiramate and nitrosi, with seizure control. Four cases were treated with phenobarbital alone, and the seizure was controlled by oxcarbazepine after the recurrence of epilepsy.

According to GBD-DCPP classification, 78 cases (60.00%) had no obvious neurological sequelae, and 52 cases (40.0%) had neurological sequelae. Among the 52 cases, 34 cases were mild sequelae and 18 cases were severe sequelae, of which eight cases were epilepsy combined with cognitive motor disorder.

## Discussion

Epilepsy seizures are common neurological complication during ALL treatment. In this study, the incidence of epileptic seizures was 5.2% in ALL children. The related studies reported that the incidence of epileptic seizures in ALL children receiving different regimens was 8 ~ 13% (12–14). The incidence of this study was relatively low. The reason may be that there was no head radiotherapy in the treatment plan, and support treatment was strengthened to improve the prognosis of children. It is reported that seizures in children with ALL usually occur during chemotherapy induction period (12). In this study, 24.02% of ALL children occurred in the induction period of chemotherapy, which may be related to the neurotoxicity of chemotherapy drugs used in ALL treatment, and the latter was the main reason for chemotherapy-related neurotoxicity. A 13 year old ALL patient was reported to have recurrent seizures after MTX treatment (15). A 11 year old ALL patient developed generalized seizures after treatment with vincristine (16). During treatment, the patient did not receive intrathecal injection of MTX or subcutaneous injection of L-asparaginase. In this study, the chemotherapy regimens used in children were basically the same, and the drug difference was not obvious. The brain organic causes such as intracranial hemorrhage, cerebral infarction, intracranial infection, and central nervous system leukemia were excluded, and the neurotoxicity of chemotherapy drugs was considered to lead to seizures. In the late stage of bone marrow transplantation, there were 49 cases (21.40%) of ALL with seizures, excluding brain organic causes, and also considering the neurotoxicity of anti-rejection drugs such as cyclooxygenase A and tacrolimus after transplantation (17). All in all, seizures in childhood acute lymphoblastic leukemia are closely related to combined chemotherapy, especially the two drugs, rather than leukemia itself.

Due to the limitation of diagnostic techniques and the complexity of children's condition, it was difficult to analyze the etiology of acute lymphoblastic leukemia epileptic seizures in the early stage. With the development of diagnostic techniques and the deepening understanding of the toxicity of chemotherapy drugs, most cases have been diagnosed more clearly through a series of neuroimaging and laboratory tests, combined with the research results in the literature. In this study, 185 out of 229 ALL children with first seizure were able to identify the etiology, among which PRES, MTX-related demyelinating encephalopathy and so on were common, which were roughly consistent with the literature (18). This study found that in the clinical manifestations associated with the first seizure in ALL children, 34.50% of the children had convulsion symptoms, and 13.54% had blurred vision symptoms. The accompanying manifestations of epilepsy are related to the etiology of epilepsy. Intracranial hemorrhage can cause disturbance of consciousness, limb movement disorder, headache with nausea or vomiting; cerebral infarction can cause movement disorder or muscle tension change; MTX — related demyelinating encephalopathy can cause generalized seizures; MTX—related demyelinating encephalopathy and PRES may have disturbance of consciousness, language, blurred vision and characteristic transient neuroimaging changes (19). In this study, due to the relatively lack of specificity of symptoms, clinical diagnosis was mainly based on neuroimaging results and the sensitivity of medical staff to the disease on the basis of excluding other organic brain damage.

The mechanism of epilepsy in children with ALL after treatment is complex and diverse. The increase of plasma homocysteine level after high dose methotrexate treatment may also be the cause of epilepsy (20). Plasma protein depletion during coagulation and fibrinolysis may occur in children receiving L-asparaginase treatment, resulting in hemorrhagic and thrombotic cerebrovascular diseases. Vincristine acts on the hypothalamus, causing excessive secretion of antidiuretic hormone leading to hyponatremia, thereby inducing seizures. Another common early complication in ALL treatment is reversible posterior encephalopathy syndrome (21), and seizures are the most common symptoms. PRES cases mainly occurred in the induction remission period of leukemia treatment, which was consistent with the results of other large sample studies. Children received multi-drug combination chemotherapy during induction remission, so it is difficult to determine which drug induced PRES. Glucocorticoid can increase blood pressure, which may promote the pathogenesis of PRES. In the literature, a case of PRES in a female patient aged 4 years old during the treatment of asthma attack with hormones was reported (22). In addition, the occurrence of vincristine, amifostine and other common drugs in the induction period and renal insufficiency can lead to hypertension, which is more likely to be accompanied by PRES (23).

In this study, 78 children with epilepsy were diagnosed as MTX—related demyelinating encephalopathy, of which 48 cases occurred after intrathecal injection of 3 ~ 9 doses of MTX, and 17 cases had chronic and irreversible brain damage after 1 ~ 6.5 years of diagnosis of ALL. Demyelinated encephalopathy is the most common neurological complication caused by MTX. The lesions mainly occur in the myelin sheath. The clinical manifestations and prognosis are different and have certain reversibility, which are generally divided into subacute and chronic demyelinated encephalopathy. Subacute encephalopathy (SAL) usually occurs within 5 ~ 14 days after intrathecal or high-dose intravenous injection of MTX. Although SAL occurs after the injection of the first dose of MTX, most patients only develop SAL after intravenous or intrathecal injection of three doses (23). SAL is most common in children over 10 years old, and can also occur in adults. According to statistics, 10 ~ 56% of patients will have SAL again, but most neurological symptoms disappear within 1 ~ 7 days after the attack. Therefore, patients can generally recover high-dose chemotherapy without leaving long-term symptoms (23). Due to the lack of histopathological data, the current research is based on the results of cerebrospinal fluid test and neuroimaging. Cerebrospinal fluid examination rarely found abnormalities, characterized by nonspecific diffusion or focal slow wave (24). In addition to acute brain dysfunction, it can also lead to chronic nervous system damage. Chronic encephalopathy (CLE) has been reported in many literature, but the understanding of the disease is still very limited. Children under 10 are more likely to occur. Different from the subsequent onset of SAL, chronic encephalopathy occurs months to years after treatment, without any obvious symptoms before. The course of disease gradually deteriorated, showing cognitive impairment, and finally caused serious damage to the nervous system, delirium, seizures, pyramidal tract signs, visual impairment and even coma (25). Histopathological studies showed that demyelination, nerve cell necrosis, and astrocytic proliferation occurred in the brain of patients, forming spongy tissue. Scanning showed brain atrophy, white matter diffuse high density, ventricular enlargement, cortical calcification (25). In this study, 17 patients with epileptic seizures in ALL years to 6.5 years all had organic damage such as brain calcification, cortical atrophy and ventricular enlargement, among which people had a history of craniocerebral radiotherapy before seizures. The occurrence of calcification and epilepsy is the performance of the late progress. If radiation encephalopathy occurs after radiotherapy, it can predict the occurrence of radiation encephalopathy. Studies have shown that the most important risk factor for the disease is brain radiotherapy before injection (25). This may be due to the destruction of the blood brain barrier by radiotherapy, so that the drug concentration into the brain tissue is too high. Hormone therapy can alleviate the disease to a certain extent, but the disease is usually irreversible.

The seizures associated with ALL are usually acute, and most seizures disappear within 24 h. In most cases, epileptic seizures during chemotherapy do not need to be diagnosed as epilepsy, but some children cannot well control seizures after the first epileptic seizure in the acute phase, or have severe brain injury, which can be secondary to symptomatic epilepsy, and need long-term antiepileptic drugs. For children with brain injury or neurological abnormalities, antiepileptic therapy is recommended for at least 2 years or until the end of leukemia treatment (7). Combined with the clinical manifestations and imaging examination of epilepsy can identify the etiology of most children. If there is no abnormality in imaging examination, even if ALL children have acute seizures, they can continue to observe after correcting or eliminating the causes, without using antiepileptic drugs. When evaluating whether antiepileptic drugs are needed to prevent seizures, clinical manifestations, imaging structural lesions and EEG epileptic discharges are important predictors of recurrent seizures, especially recurrent seizures.

In this study, the brain images of ALL children with epileptic seizures were less analyzed. And follow-up sample size is too small, need to follow up more center large sample clinical research in the future, in order to accurately determine the prognosis of ALL children with epilepsy.

## Conclusion

Intensive treatment of acute lymphoblastic leukemia has been very mature, and basically no brain radiotherapy, largely reducing the long-term damage to the central nervous system. However, the disadvantage is that the incidence of acute central nervous poisoning increased significantly during treatment. Now, drug-induced neurological damage is more common than leukemia central nervous system infiltration, and also fatal. Most of the acute iatrogenic damage in the treatment of childhood leukemia is reversible, but its neurological symptoms are often lack of specificity. Therefore, it is more necessary for doctors in neurology, radiology and hematology to cooperate closely, so as to find out the pathogenic factors in time, make patients get early diagnosis, and avoid long-term systemic damage and unnecessary treatment.

## Data Availability Statement

The original contributions presented in the study are included in the article/supplementary material, further inquiries can be directed to the corresponding author/s.

## Ethics Statement

The studies involving human participants were reviewed and approved by Ethics Committee of Children's Hospital of Soochow University. Written informed consent to participate in this study was provided by the participants' legal guardian/next of kin.

## Author Contributions

RL and J-HT: conception design of the research and critical revision of the manuscript for intellectual content. RL, J-HT, B-BZ, and Y-YD: acquisition of data. RL, X-YS, and RQ: analysis and interpretation of the data. Obtaining financing: none. RL: writing of the manuscript and statistical analysis. All authors contributed to the article and approved the submitted version.

## Funding

This work was supported by the Science and Technology Project of the Health and Health Committee of Jiangsu Province (No. H2018010). The funder of the study had no role in the study design, collection, analysis, and interpretation of data, or writing of this manuscript.

## Conflict of Interest

The authors declare that the research was conducted in the absence of any commercial or financial relationships that could be construed as a potential conflict of interest.

## Publisher's Note

All claims expressed in this article are solely those of the authors and do not necessarily represent those of their affiliated organizations, or those of the publisher, the editors and the reviewers. Any product that may be evaluated in this article, or claim that may be made by its manufacturer, is not guaranteed or endorsed by the publisher.

## References

[1] Hunger SP Loh ML Whitlock JA Winick NJ Carroll WL Devidas M. Acute Lymphoblastic Leukemia Committee. Children's Oncology Group's 2013 blueprint for research: acute lymphoblastic leukemia. Pediatr Blood Cancer. (2013) 60:957–63. 10.1002/pbc.24420 23255467 PMC4045498

[2] Kaprin AD Starinskij VV Petrov GV. Malignant neoplasms in Russia in 2015 (morbidity and mortality). Moscow: Herzen MORI-branch of the NMRRC of the Ministry of Health of the Russian Federation. (2017). p. 1–250.

[3] Xiru Y Kelly P. Progress in diagnosis and treatment of central nervous system leukemia in children. Int J Pediatr. (2016) 43:307–10. 10.3760/cma.j.issn

[4] Maxwell RR Cole PD. Pharmacogenetic predictors of treatment-related toxicity among children with acute lymphoblastic leukemia. Curr Hematol Malig Rep. (2017) 12:176–86. 10.1007/s11899-017-0376-z 28317081

[5] Tang JH Tian JM Sheng M Hu SY Li Y Zhang LY. Study of posterior reversible encephalopathy syndrome in children with acute lymphoblastic leukemia after induction chemotherapy. J Child Neurol. (2016) 31:279–84. 10.1177/0883073815589758 26060305

[6] Goyal M Bangert BA Wiznitzer M. Mesial temporal sclerosis in acute childhood leukemias. Epilepsia. (2003) 44:131–4. 10.1046/j.1528-1157.2003.20302.x 12581241

[7] Anastasopoulou S Heyman M Eriksson MA Niinimäki R Taskinen M Mikkel S. Seizures during treatment of childhood acute lymphoblastic leukemia: a population-based cohort study. Eur J Paediatr Neurol. (2020) 27:72–7. 10.1016/j.ejpn.2020.04.004 32340855

[8] Bo C Ying X Yongchun S Xianhao W Xianmin G Qicheng Z. A clinical study on side effects of CCLGALL 08 regimen in the treatment of children with acute lymphoblastic leukemia. Chin J Contemp Pediatr. (2013) 15:737–42. 10.7499/j.issn.1008-8830.2013.09.007 24034915

[9] Fengling X Xianmin G Xianhao W Yali S Jianwen Xiao Yuxia Guo. Clinical analysis of severe adverse reactions associated with chemotherapy in children with acute lymphoblastic leukemia. Chin J Contemp Pediatr. (2020) 22:828–33. 10.7499/j.issn.1008-8830.2003253 32800028 PMC7441514

[10] Fisher RS Acevedo C Arzimanoglou A Bogacz A Cross JH Elger CE. ILAE official report: a practical clinical definition of epilepsy. Epilepsia. (2014) 55:475–82. 10.1111/epi.12550 24730690

[11] Chinese Society of Neurology; Chinese Society of Electroence phalography and Epilepsy. Interpretation by chinese experts of the new classification of seizures by the international league against epilepsy. Chin J Neurol. (2019) 52:977–80. 10.3760/cma.j.issn.1006-7876.2019.11.021

[12] Millan NC Pastrana A Guitter MR Zubizarreta PA Monges MS Felice MS. Acute and sub-acute neurological toxicity in children treated for acute lymphoblastic leukemia. Leuk Res. (2018) 65:86–93. 10.1016/j.leukres.2017.12.010 29328996

[13] Nassar SL Conklin HM Zhou Y Ashford JM Reddick WE Glass JO. Neurocognitive outcomes among children who experienced seizures during treatment for acute lymphoblastic leukemia. Pediatr Blood Cancer. (2017) 64:10. 10.1002/pbc.26436 28130818 PMC5469699

[14] Andrés-Jensen L Attarbaschi A Bardi E Barzilai-Birenboim S Bhojwani D Hagleitner MM. Severe Toxicity Working Group. Severe toxicity free survival: physician-derived definitions of unacceptable long-term toxicities following acute lymphocytic leukemia. Lancet Haematol. (2021) 8:e513–23. 10.1016/S2352-3026(21)00136-8 34171282

[15] Hamamoto K Oriuchi N Kanazawa T Higuchi T Endo K. Mesial temporal sclerosis associated with methotrexate-induced leukoencephalopathy. Pediatr Neurol. (2009) 40:306–9. 10.1016/j.pediatrneurol.2008.10.022 19302946

[16] Mahapatra M Kumar R Choudhry VP. Seizures as an adverse drug reaction after therapeutic dose of vincristine. Ann Hematol. (2007) 86:153–4. 10.1007/s00277-006-0201-6 17031686

[17] Liu SX Xiao PF Hu SY He HL Li J Lu J. Clinical analysis of nervous system complications after hematopoietic stem cell transplantation in children. Chin J Organ Transplant. (2019) 11:691–5. 10.3760/cma.j.issn.0254-1785.2019.11.010

[18] Antunes NL. Seizures in children with systemic cancer. Pediatr Neurol. (2003) 28:190–3. 10.1016/S0887-8994(02)00508-8 12770671

[19] Ando Y Ono Y Sano A Fujita N Ono S. Posterior reversible encephalopathy syndrome: a review of the literature. Intern Med. (2022) 61:135–41. 10.2169/internalmedicine.7520-21 34275982 PMC8851194

[20] Kishi S Griener J Cheng C Das S Cook EH Pei D. Homocysteine, pharmacogenetics, and neurotoxicity in children with leukemia. J Clin Oncol. (2003) 21:3084–91. 10.1200/JCO.2003.07.056 12915598

[21] Banerjee JS Heyman M Palomäki M Lähteenmäki P Arola M Riikonen PV. Posterior reversible encephalopathy syndrome: risk factors and impact on the outcome in children with acute lymphoblastic leukemia treated with nordic protocols. J Pediatr Hematol Oncol. (2018) 40:e13–8. 10.1097/MPH.0000000000001009 29200159

[22] Kurahashi H Okumura A Koide T Ando Y Hirata H Magota M. Posterior reversible encephalopathy syndrome in a child with bronchial asthma. Brain Dev. (2006) 28:544–6. 10.1016/j.braindev.2006.02.008 16617001

[23] de Laat P Te Winkel ML Devos AS Catsman-Berrevoets CE Pieters R van den Heuvel-Eibrink MM. Posterior reversible encephalopathy syndrome in childhood cancer. Ann Oncol. (2011) 22:472–8. 10.1093/annonc/mdq382 20699277

[24] Salkade PR Lim TA. Methotrexate-induced acute toxic leukoencephalopathy. J Cancer Res Ther. (2012) 8:292–6. 10.4103/0973-1482.98993 22842379

[25] Inaba H Khan RB Laningham FH Crews KR Pui CH Daw NC. Clinical and radiological characteristics of methotrexate-induced acute encephalopathy in pediatric patients with cancer. Ann Oncol. (2008) 19:178–84. 10.1093/annonc/mdm466 17947226

